# Bone morphogenetic protein 6 induces downregulation of pentraxin 3 expression in human granulosa lutein cells in women with polycystic ovary syndrome

**DOI:** 10.1007/s10815-023-02972-z

**Published:** 2023-11-06

**Authors:** Xin Xin, Hsun-Ming Chang, Peter C. K. Leung, Li Dong, Jiaxi Li, Fang Lian, Haicui Wu

**Affiliations:** 1 First School of Clinical Medicine, Shandong University of Traditional Chinese Medicine, Jinan, 250011 China; 2 Reproductive Medicine Center, Department of Obstetrics and Gynecology, China Medical University Hospital, Taichung, Taiwan; 3 Department of Obstetrics and Gynaecology, BC Children’s Hospital Research Institute, University of British Columbia, Vancouver, British Columbia Canada; 4 Affiliated Hospital of Shandong University of Traditional Chinese Medicine, Jinan, 250014 China

**Keywords:** Polycystic ovary syndrome, Bone morphogenetic protein 6, Pentraxin 3, SMAD1/5/8, ALK2/3/6, Human granulosa-lutein cells

## Abstract

**Purpose:**

To evaluate whether PTX3 is differentially expressed in the granulosa lutein cells derived from women with PCOS and whether BMP6 can regulate the expression of PTX3 in hGL cells.

**Methods:**

The expression levels of BMP6 and PTX3 in granulosa lutein cells were evaluated by RT-qPCR. The correlation between the expression levels of BMP6 /PTX3 and oocyte quality indexes were analyzed using clinical samples. The cells were incubated with BMP6 at different concentrations and times to check the expression of PTX3 in KGN cells. TGF-β type I inhibitors and small interfering RNA targeting ALK2/3/6,SMAD1/5/8 and SMAD4 were used to study the involvement of SMAD dependent pathways in KGN cells.

**Results:**

The levels of BMP6 in hGL cells were negatively correlated with the corresponding oocyte maturation rate and high-quality embryo rate, whereas the levels of PTX3 were positively correlated with the corresponding oocyte maturation rate in PCOS. Additionally, the in vitro cell cultured results showed BMP6 significantly inhibited the expression of PTX3 in KGN cells. Furthermore, using a dual inhibition approach (kinase inhibitors and small interfering RNAs), we identified the ALK2/ALK3 type I receptors and BMPR2/ACVR2A type II receptors and the downstream SMAD1/SMAD5-SMAD4 signaling pathway were responsible for the BMP6-induced cellular activities in KGN cells.

**Conclusions:**

The suppressive effect of BMP6 on PTX3 was mediated by ALK2/ALK3 type I receptors and BMPR2/ACVR2A type II receptors in granulosa cells through the SMAD1/5-SMAD4 dependent signaling pathway in PCOS.Our findings provides new insights into the understanding of the pathogenesis of PCOS-related ovulatory disorders.

## Introduction

Ovulation is an essential event for mammals to maintain normal reproductive activities. The dysregulation of ovulation is one of the leading causes of female infertility. Polycystic ovary syndrome (PCOS) is one of the most common causes of female infertility due to ovulation disorders and because the etiology of PCOS is currently unclear, it is one of the most challenging research topics in the field of female reproduction. In humans, ovulation is regulated by gonadotropins and involves a series of steps, such as oocyte meiosis restart, cumulus-oocyte complex (COC) formation, extracellular matrix reconstruction, and COC expansion [1]. This series of processes is essential for successful fertilization and early embryo development after the entrance of the oocyte into the oviduct. More importantly, successful ovulation largely depends on the proper expansion of COC under the control of gonadotropins and several growth factors [2]. COC expansion is initiated by the accumulation of hyaluronic acid derived from granulosa cells; this process plays an important role in regulating oocyte maturation and ovulation. Moreover, the extent of COC expansion is a crucial morphological indicator for oocyte maturation and embryo quality in clinical assessment [3].

Pentraxin 3 (PTX3) belongs to the pentraxin superfamily and is expressed in various human tissues. As a multifunctional secreted protein, PTX3 regulates cell proliferation, angiogenesis, migration, and invasion and plays a vital role in the regulation of immune and inflammatory responses, matrix deposition, and female reproduction [4]. PTX3 is a key factor in the formation of a stable hyaluronic acid structure, which is essential for mammalian ovulatory function [5]. The expression of PTX3 in granulosa cells is closely associated with oocyte quality and subsequent fertilization rate [6]. Gene inactivation of PTX3 decreases the fertilization ability of oocytes by destroying the structural integrity of COC. Zhang et al. found that the expression level of PTX3 in cumulus cells is closely related to the fertilization ability of the corresponding oocyte and the subsequent development of an embryo with implantation potential [7]. An animal study using transcriptional analysis indicated that the oocytes with higher expressions of PTX3 had higher potential for division and fertilization in buffalo [8]. Similarly, the downregulation of PTX3 expression in granulosa cells resulted in a defect in female fertility [9]. Most studies suggested that the plasma level of PTX3 is associated with the pathogenesis of obesity, low-grade inflammation, and insulin resistance in patients with PCOS. However, the molecular mechanisms underlying the correlation between PTX3 level and PCOS [10–12] have not been determined. Considering the valuable role of PTX3 in follicular development and ovulation, relevant research regarding PTX3 modulation may help find new targets for managing ovulation dysfunction. To our knowledge, only a few studies on PTX3 levels in follicular fluid samples and granulosa cells obtained from patients with PCOS have been carried out.

Bone morphogenetic proteins (BMPs) are the largest members of transforming growth factor β (TGF-β) superfamily growth factors and are extremely important for maintaining female reproduction. Previous studies have shown that several BMPs, including BMP2, BMP4, BMP6, and BMP7 can regulate hyaluronic acid synthesis in human granulosa cells, leading to ovulation [13–15]. BMPs phosphorylate and activate intracellular signaling mediators Sma- and Mad-related protein (SMAD)1/5/8 after binding to the functional receptors, TGF-β type I and type II receptors, on the cell membrane [16]. In particular, BMP6 has been shown to regulate mammalian follicle function, oocyte maturation, and luteal function [17, 18] by modulating intercellular communication in the ovary [19]. Additionally, the results obtained from clinical studies have revealed that the dysregulation of BMP6 signaling in the ovary is related to the pathogenesis of PCOS and ovulatory dysfunction [20]. Taken together, BMP6 is an essential intraovarian regulator for maintaining normal follicular development.

In summary, both PTX3 and BMP6 play a role in the hyaluronic acid structure, affecting cumulus expansion and leading to follicular development disorders, which may be related to the occurrence of PCOS. However, the presence of an interaction and regulatory relationship between PTX3 and BMP6 and the significance of this interaction in the occurrence and development of PCOS remain unknown and have great exploratory value.

This study was proposed to evaluate the association between the expression of BMP6 and PTX3 in the human granulosa-lutein (hGL cells) and PCOS through both clinical observation and in vitro cell culture experiments. By studying the molecular mechanism by which BMP6 regulates the expression of PTX3 in hGL cells, we aimed to understand the regulatory process of the ovulation event and identify new targets for treating ovulation disorders at the molecular level.

## Materials and methods

### Patients included in this study

All participants underwent the initial cycle of in vitro fertilization/embryo transfer (IVF/ET) at the reproductive and genetic center of Shandong University of Traditional Chinese Medicine from January 2018 to January 2022. The calculation of the sample size was performed before the start of the study. Our study was considered a pilot, which aimed to gather at least 12–25 participants per group [21]. The participants included 31 women with PCOS (as a study group) and 31 patients with tubal factor infertility (as a control group). The diagnosis of PCOS was primarily based on the criteria established by the Rotterdam Society of Human Reproduction and Embryology/American Society of Reproductive Medicine, revised in 2003 [22]. All participants had no other endocrine system diseases, genital malformations, or chromosomal disorders and had not taken hormones or addictive drugs recently. The recruitment criteria for the PCOS group and the control group were similar, and participants in both groups had similar characteristics, including age, sex, and physical condition other than PCOS, to overcome selection bias [23].

All participants provided written informed consent, and the study was approved by the ethics committee of the Reproductive and Genetic Center of Shandong University of Traditional Chinese Medicine, approval no. SDTCM/E2110-03. All procedures performed involving human participants met the ethical standards of the institutions, national research committees, and 1964 Declaration of Helsinki and its subsequent amendments or similar ethical standards.

### Ovarian stimulation and oocyte retrieval

Patients undergoing IVF/ET treatment initially received controlled ovarian hyperstimulation using medication that prevents the premature luteinization, including GnRH antagonists (ganirelix, Merck, Canada) in the follicular phase or triptorelin acetate (Synarel, Pfizer, Canada) in the luteal phase of the previous cycle to downregulate the pituitary gonadotropin-releasing hormone receptors and subsequent transduction pathways. On the second day of the menstrual cycle, appropriate human menopausal gonadotropin (hMG, Menopur, Ferring, Canada) or recombinant FSH (Puregon, Merck, Canada) were administered to stimulate follicular growth. When the diameter of the leading follicle reached > 18 mm or the diameters of at least three follicles reached > 17 mm, hCG (Pregnyl, Merck) was administered to trigger final oocyte maturation. Oocytes were retrieved under vaginal ultrasound-guiding 34–36 h after the trigger, and corresponding follicular fluid was collected.

### Oocyte scoring criteria

The embryologists collected all the retrieved oocytes and evaluated the maturation of oocytes based on the following four perspectives [24]:

1. Cumulus size: large cumulus cells and loose arrangement (1 point); small or clustered cumulus cells and close arrangement (0 points).
2. Oocyte color and transparency: light and good transparency (1 point); dark and dim transparency (0 points).
3. Radial crown arrangement: radial crown cells arranged radially (1 point); radial crown cells did not disperse, or the radial crown was too divergent (0 points).
4. Oocyte visibility: the oocyte was visible under the microscope (1 point); the oocyte visibility was fuzzy and light in color (0 points).

According to the aforementioned scoring standards, oocytes with scores of 3–4 were considered of high quality.

### In vitro fertilization and embryo culture

Fertilization was determined by observing the formation of the pronucleus after 17 h of insemination. When two different pronuclei containing nuclei (2 PN) were observed, successful fertilization was determined [25]. The morphology and quality of embryos were evaluated based on the cleavage of cells after 72 h. Grade I embryos included 7–9 cells with uniform germ layers and fragment rates of < 10%. The fragment rate of grade II embryos was between 11 and 25%. The cell division of grade III embryos was irregular, and the fragmentation rate exceeded 25% [26]. Grade I embryos were considered to be high-quality embryos. Grade I and II embryos were considered viable. Viable embryos were used to perform fresh embryo transfers or frozen-thawed embryo transfers.

### Clinical outcomes

The oocyte maturation rate was defined as the number of oocytes that scored 3–4 divided by the total number of oocytes retrieved. The fertilization rate was defined as the number of fertilized oocytes divided by the total number of retrieved oocytes. The cleavage rate was defined as the number of cleaved embryos divided by the total number of fertilized eggs. The viable embryo rate was defined as the number of embryos available for transfer divided by the number of all cleaved embryos. The high-quality embryo rate was defined as the number of high-quality embryos divided by the number of all viable embryos. Clinical pregnancy was defined as the presence of a pregnancy sac and a fetal pole in the uterine cavity monitored by ultrasound at six weeks of gestation. The cumulative clinical pregnancy rate was defined as the number of clinical pregnancies divided by the total number of all transferred cycles. The main outcomes were measured by the high-quality oocyte rate and cumulative clinical pregnancy rate. The secondary outcomes were measured by the fertilization rate, cleavage rate, transferred embryo rate, and high-quality embryo rate.

### Human ovarian granulosa cell line (KGN cells) culture

The human ovarian granulosa cell line (KGN cells) is a commonly used cell model for in vitro experiments to study ovarian functions because these cells are easy to isolate, culture, and transfect and can be analyzed by immunocytochemical methods [27, 28]. KGN cells were seeded (2–4 × 105 per well) in six-well plates and were cultured at 37 ºC in 5% CO_2_ and 95% air. Dulbecco's Modified Eagle's Medium/Nutrient Mixture F-12 Ham (DMEM/F-12; Sigma Aldrich, USA) was used to culture the cells and was supplemented with penicillin (100 U/mL, Invitrogen, Life Technologies, USA), streptomycin sulfate (100 μG/mL, Invitrogen, Life Technologies, USA), glutamine (1x, Invitrogen, Life Technologies, USA), and 10% carbon/dextran treated fetal bovine serum (10%, HyClone, USA). The medium was changed every other day. All cells were resuspended in a serum-free medium for 24 h before the experiment. For concentration-dependent studies, cells were treated with BMP6 at different concentrations (1, 10, or 100 ng/mL) for 24 h. For time course studies, cells were treated with 100 ng/mL BMP6 for 3, 6, 12, or 24 h. Cells were harvested for reverse transcription-quantitative polymerase chain reaction (RT‒qPCR) and Western blot analysis to examine messenger RNA (mRNA) and protein levels, respectively. The PTX3 levels secreted in the culture medium were examined using an enzyme immunoassay.

### Preparation and culture of primary hGL cells

The primary hGL cells used in the experiment were all isolated from the remaining follicular fluid obtained from patients undergoing IVF treatment at the reproductive and genetic center of the affiliated hospital of Shandong University of Traditional Chinese Medicine. The hGL cells were centrifuged and purified by density gradient centrifugation precipitation after collecting follicular fluid [29]. These cells were counted with a hemocytometer, and cell viability was assessed by trypan blue (0.04%) exclusion. Purified primary hGL cells were seeded (2 × 10^5^ cells per well in 12-well plates) and cultured in a humidified atmosphere of 5% CO_2_ and 95% air at 37 ºC. Cells were cultured in DMEM/DMEM/F-12 (Sigma-Aldrich Corp., Oakville, ON) supplemented with 10% fetal bovine serum (Hyclone, Logan, UT), 100 U/mL penicillin (Life Technologies, Inc./BRL, Grand Island, NY), 100 μg/mL streptomycin sulfate (Life Technologies), and 1 × GlutaMAX (Life Technologies). The culture medium was changed every other day. All cells were resuspended in a serum-free medium for 24 h before the experiment.

### Antibodies and reagents (Table 1)

**Table 1:** Antibodies and reagents

Name	Company
Recombinant human BMP6 protein (507-BP)	R&D Systems (Minneapolis, MN, USA)
Dorsomorphin dihydrochloride (DM) (3093)	
4-[6-[4-(1-methylethoxy) phenyl] pyrazolo[1,5-a] pyrimidin-3-yl]-quinoline DMH-1 (DMH-1) (4126) antibody	
TGF-β type I receptor inhibitor SB431542	Sigma Aldrich (St Louis, MO, USA)
Polyclonal rabbit anti-phospho-SMAD1 (Ser463/465) antibody	Cell Signaling Technology (Beverly, MA)
Polyclonal rabbit anti-phospho-SMAD5 (Ser463/465) antibody	
Polyclonal rabbit anti-phospho-SMAD8 (Ser426/428) antibody (13,820, diluted at 1:1000)	
Olyclonal rabbit anti-SMAD4 (9515, diluted at 1:1000) antibody	
Horseradish peroxidase-conjugated goat anti-rabbit (diluted at 1:5000)	Bio-Rad Laboratories (Hercules, CA)
Goat anti-mouse IgGs (diluted at 1:5000)	
Horseradish peroxidase-conjugated donkey anti-goat IgG	Santa Cruz Biotechnology

### Reverse transcription quantitative real-time PCR (RT‒qPCR)

Cells were washed with cold phosphate buffered saline (PBS), and total RNA was extracted from hGL cells using TRIzol Reagent (Invitrogen, Life Technologies) according to the manufacturer's instructions. Typically, 2 μg of RNA was used to produce first-strand cDNA with random primers and Moloney murine leukemia virus (MMLV) reverse transcriptase (Promega, Madison, USA). Every 20-μL mixture contained 10 μL of 1X SYBR Green PCR Master Mix (Applied Biosystems, USA), 20 ng of cDNA template, and 250 nm of primer. The reverse transcription reaction conditions were as follows: 42 ºC for 40 min and 65 ºC for 10 min. After the reaction, cDNA was obtained and stored at − 80 ºC. The primer sequences used in this study are listed in Table 2. The primers used for the TaqMan gene expression assays were as follows: PTX3 (Hs00173615_m1), ACVR1 (ALK2, Hs00153836_m1), BMPR1A (ALK3, Hs01034913_g1), BMPR1B (ALK6, Hs01010965_m1), SMAD1 (Hs01077084_m1); SMAD5 (Hs00195437_m1), SMAD8 (HS001195441_m1), SMAD4 (Hs00929647_m1), and GAPDH (Hs02758991_G2) (Applied Biosystems, Foster, CA). PCR was performed using the Applied Biosystems 7300 real-time fluorescent quantitative PCR system. The RT-qPCR program and system conditions were as follows: 95 ºC for 3 min, followed by 95 ºC for 10 s and 60 ºC for 30 s, all for a total of 35 cycles. Three independent experiments were performed using different cultures, and each sample was repeated three times. Relative quantitative analysis of mRNA levels was performed using the comparative cycle threshold (CT) method, with GAPDH as a reference gene and the calculation formula 2^−∆∆Ct^. All primers used in this study passed the validation test.

**Table 2:** Primer synthesis information

Gene Number	Primers	Sequence (5’-3’)
Reference	GAPDH-F	GAGTCACGGATCAAGATTGGTCGT
	GAPDH-R	GACAGACTTCTCTCTCTGTTCGTCTCAG
1	PTX3-F	TCTCTGGTCTGCAGTGTGG
	PTX3-R	TGAAGAGCTTCCCATTCC
2	SMAD4-F	TGGCCCAGGATCAGTAGGT
	SMAD4-R	CATCAACAATTCCAGCA

### Western blot analysis

After washing with cold PBS, the cells were lysed in lysis buffer (Cell Signaling) containing a protease inhibitor cocktail (Sigma‒Aldrich). The cell lysates were centrifuged at 14,000 rpm for 15 min at 4 ℃, and the supernatants were subsequently collected. The protein concentration in the supernatant was quantitatively determined using the DC protein assay (Bio Rad Laboratories). Equal amounts of protein were separated using 10% sodium dodecyl sulfate‒polyacrylamide gel electrophoresis (SDS‒PAGE) and transferred to a polyvinylidene fluoride membrane. After 1 h of blocking with 5% nonfat dry milk in TBS buffer, the membranes were incubated overnight with relevant primary antibodies and washed three times with TBST buffer (TBS with Tween-20); the membranes were incubated for 1 h with appropriate peroxidase-conjugated secondary antibodies. The immunoreactive bands were detected by enhanced chemiluminescent substrate or SuperSignal West Femto Chemiluminescent Substrate (Pierce, Rockford, IL). The membranes were stripped with stripping buffer at 50 ºC for 30 min and reprobed with rabbit anti-SMAD1/5/8 or mouse anti-α-tubulin antibody as a loading control.

### Small interfering RNA (siRNA) transfection

We used 25 nM of ON-TARGETplus SMARTpool or 25 nM ON-TARGETplus Non-Targeting Pool (Thermo Fisher Scientific; Lafayette, CO, USA) to decrease the level of ALK2, ALK3, ALK6, BMPR2, ACVR2A, ACVRR2B, SMAD1, SMAD5, SMAD8, or SMAD4 expression. Cells were cultured to 50% confluence in antibiotic-free DMEM/F12 medium containing 10% FBS and then transfected with 25 nM siRNA for 48 h using Lipofectamine RNAiMAX (13,778–150; Invitrogen, Life Technologies). SiCONTROL Non-Targeting Pool siRNA was used as the transfection control. The knockdown efficiency was confirmed by real-time quantitative RT-PCR or Western blot analysis.

### Measurement of BMP6 and PTX3 by enzyme-linked immunosorbent assay

The cell culture medium was collected for the enzyme-linked immunosorbent assay. BMP6 and PTX3 protein production levels in culture medium or follicular fluid were measured by an enzyme immunoassay kit (R&D Systems) per the manufacturer's instructions. The levels of BMP6 and PTX3 were normalized to the protein concentration of each cell lysate. Each sample was measured three times.

### Statistical analysis

The results were analyzed using SPSS Version 22. Comparisons between the two groups were performed by Student’s t-test or the Mann‒Whitney U test for continuous variables and the χ^2^ test for categorical variables. Spearman correlation analysis was applied to identify correlations between BMP6/PTX3 expression and clinical indicators. Experimental results were presented as mean ± standard error of the mean (SEM) based on at least three independent experiments. The results were analyzed by one-way analysis of variance followed by Tukey’s multiple-comparison tests using PRISM software (GraphPad Software, San Diego, CA). Data were considered significantly different if the *P*-value was < 0.05.

## Results

### Clinical data analysis

#### Baseline characteristics of recruited patients

This study included 31 patients with PCOS and 31 patients with tubal factor infertility (with normal endocrine function). A total of 71 embryo transfer cycles were included. The baseline characteristics of patients are displayed in Table 3. No significant difference was identified in patient age, infertility duration and cause, and body mass index (BMI) between the two groups (*P* > 0.05). Additionally, no significant difference was present in the duration of gonadotropin (Gn) administration (*P* > 0.05). However, a significantly lower total dose of Gn during the stimulation cycle was found in the PCOS group than that in the control group (*P* < 0.05). Moreover, significantly higher levels of basal LH, basal testosterone, and anti-müllerian hormone (AMH) were found in the PCOS group (*P* < 0.001). The number of retrieved oocytes was also higher in the PCOS group (*P* < 0.001).

**Table 3:** Baseline characteristics of recruited patients

	PCOS group	Control group	*P*-value
No of patients	31	31	
Female age at oocyte retrieval(years)	30.10 ± 3.38	30.52 ± 3.01	0.61
Duration of infertility (years)	2.97 ± 1.78	2.74 ± 1.41	0.58
Infertility type			0.31
Primary infertility	16 (51.6%)	12 (38.7%)	
Secondary infertility	15 (48.4%)	19 (61.3%)	
Duration of COS	11.45 ± 1.65	11.74 ± 1.12	0.42
Total Gn dose administered	2804.19 ± 515.75	3174.20 ± 595.67	0.01*
AMH level (ng/ml)	6.75 ± 1.69	2.48 ± 0.91	< 0.001**
BMI (kg/m^2^)	24.19 ± 4.52	23.15 ± 3.51	0.31
Basic LH level (IU/L)	8.83 ± 4.87	4.52 ± 1.39	< 0.001**
Basic T level (ng/ml)	1.15 ± 0.45	0.34 ± 0.18	< 0.001**
No. of oocytes retrieved	24.78 ± 13.87	10.68 ± 3.11	< 0.001**

Data are presented as mean ± SD for continuous variables and n (%) for dichotomous variables. All *P* values were assessed with the use of Student’s t test or χ^2^(**p < 0.01,*p < 0.05)

#### Clinical outcomes

We conducted statistical analysis on the relevant indicators of oocyte retrieval and embryo transfer cycle of the two groups. The results are presented in Table 4. In the oocytes removed from the two groups, the high-quality oocyte rate of the PCOS group was significantly lower than that of the control group (50.9% vs. 70.1%, *P* < 0.001). Although no significant difference was identified between the fertilization rates of the two groups, the cleavage rate of the PCOS group was still significantly lower than that of the control group (59.3% vs. 74.1%, *P* < 0.001). A significantly lower viable embryo rate was identified in the PCOS group (36.8% vs. 52.7%, *P* < 0.001); however, no significant difference was determined in the high-quality embryo rates between the two groups (Table 2). Additionally, the cumulative pregnancy rate of the PCOS group was lower than that of the control group (28.6% vs. 52.8%, *P* = 0.038).

**Table 4:** Clinical outcomes

	PCOS group	Control group	*P*-value
No of patients	31	31	
Oocyte maturation rate (%)	50.9% (391/768)	70.1% (232/331)	< 0.001**
Fertilization rate (%)	67.2% (516/768)	67.7% (224/331)	0.88
Cleavage rate (%)	59.3% (306/516)	74.1% (166/224)	< 0.001**
Viable embryo rate (%)	36.8% (190/516)	52.7% (118/224)	< 0.001**
High-quality embryo rate (%)	35.3% (67/190)	28.0% (33/118)	0.18
Cumulative clinical pregnancy rate (%)	28.6% (10/35)	52.8% (19/36)	0.04*

Data are presented as mean ± SD for continuous variables and n (%) for dichotomous variables. All *P* values were assessed with the use of χ^2^(**p < 0.01,*p < 0.05)

### Increased expression of BMP6 and decreased expression of PTX3 in the cumulus granulosacells and follicular fluid obtained from patients with PCOS

**Fig. 1:**
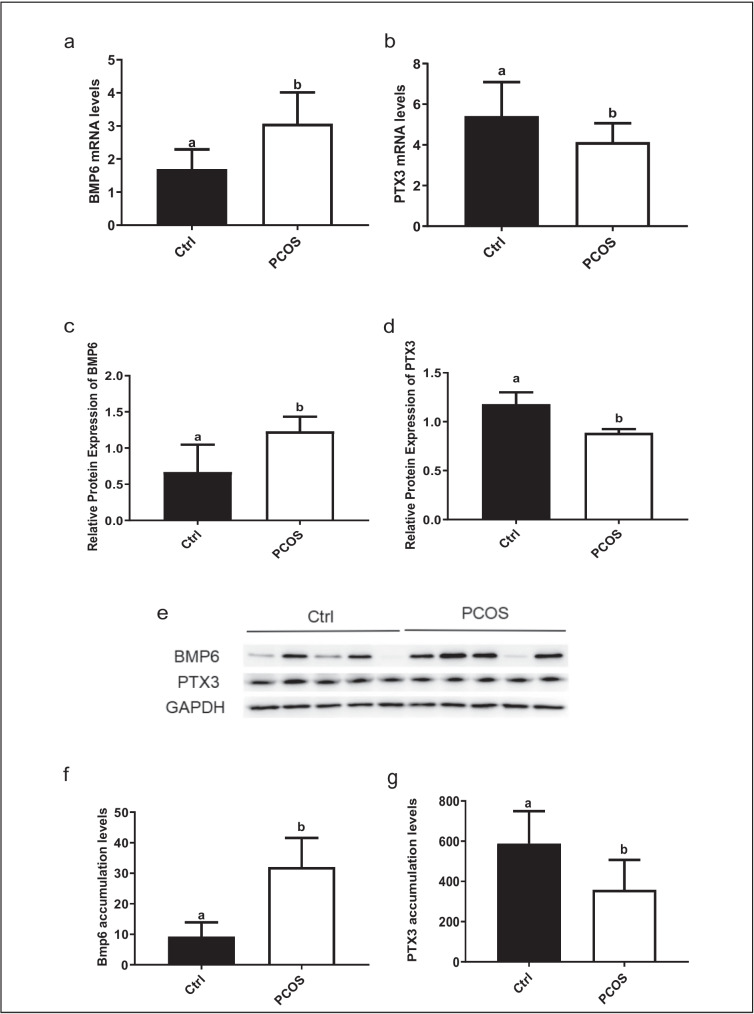
Comparison of the expression of BMP6 and PTX3 in human granulosa-lutein (hGL) cells and follicular fluid between PCOS and control groups. The isolated cells and follicular fluid were directly used for the mRNA analysis without further culture. The mRNA levels of BMP6 and PTX3 in hGL cells of patients with PCOS and control patients were examined using RT-qPCR **(a–b**). The protein levels of BMP6 and PTX3 in the granulosa cells of patients with PCOS and control patients were then examined using Western blot analysis (**c–e**). The accumulated protein levels of BMP6 and PTX3 in the follicular fluid samples obtained from patients with PCOS and control patients were examined using ELISA (**f–g**). The results were expressed as means ± standard errors of at least three independent experiments, with different letters indicating statistically significant differences (*P* < 0.05)

To determine whether the expression levels of BMP6 and PTX3 in the cumulus granulosacells of patients with PCOS differed from those of control patients, we measured the mRNA levels of BMP6 and PTX3 of cumulus granulosacells using RT-qPCR. The isolated cells and follicular fluid were directly used for the mRNA analysis without further culture. The results are presented in Fig. 1a and b, showing that BMP6 mRNA levels were upregulated, whereas PTX3 mRNA levels were downregulated in the hGL cells of patients with PCOS. Additionally, the BMP6 protein levels were increased, whereas the PTX3 protein levels were decreased in follicular fluid samples obtained from patients with PCOS, compared with those from the control group (*P* < 0.05, Fig. 1c to e). Similarly, using an enzyme-linked immunosorbent assay, we found that the BMP6 protein levels were increased, whereas the PTX3 protein levels were decreased in the hGL cells obtained from patients with PCOS, compared with those from the control group (*P* < 0.05) (Fig. 1f and g).

### Correlation between the mRNA levels of BMP6 and PTX3 in hGL cells and clinical outcomes in patients with PCOS

**Fig. 2:**
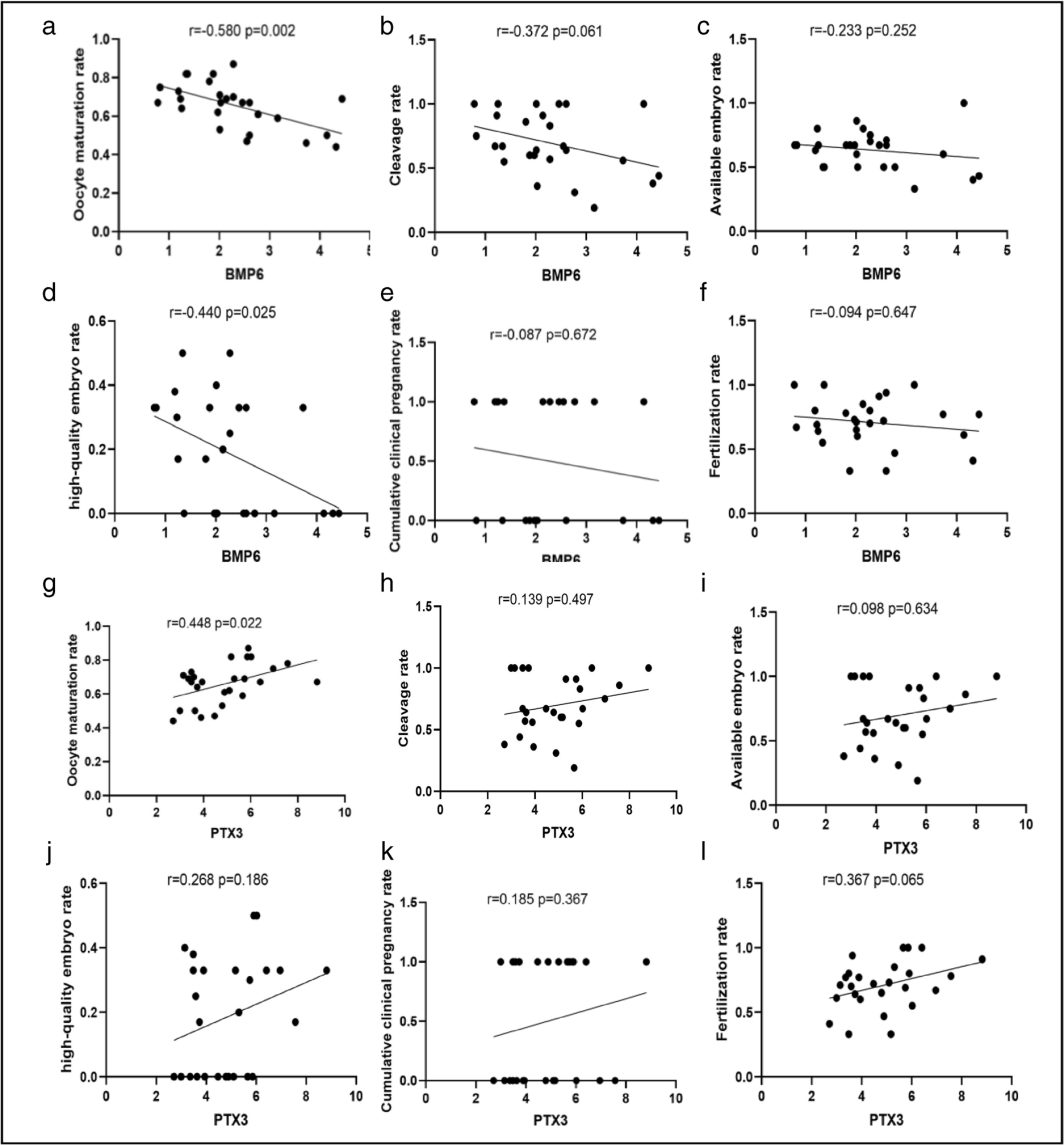
Correlation between the mRNA levels of BMP6 and PTX3 in hGL cells and clinical outcomes in patients with PCOS. Spearman correlation analyses were used to investigate the correlation between the mRNA levels of BMP6 and the oocyte maturation rate (**a**), cleavage rate (**b**), available embryo rate (**c**), high-quality embryo rate (**d**), cumulative clinical pregnancy rate (**e**), and fertilization rate (**f**) in patients with PCOS. Spearman correlation analyses were used to investigate the correlation between the mRNA levels of PTX3 and the oocyte maturation rate (**g**), cleavage rate (**h**), available embryo rate (**i**), high-quality embryo rate (**j**), cumulative clinical pregnancy rate (**k**), and fertilization rate (**l**) in patients with PCOS

To investigate the effects of the expression changes in BMP6 and PTX3 in the cumulus granulosacells of patients with PCOS on the clinical outcomes of IVF treatment, we conducted a correlation study using the Spearman correlation analysis. The results showed that the mRNA levels of BMP6 in hGL cells were negatively correlated with the oocyte maturation rate (*r* =  − 0.580, *P* < 0.01, Fig. 2a) and high-quality embryo rate (*r* =  − 0.440, *P* < 0.05, Fig. 2d). This indicates that high the level of BMP6 in hGL cells was related to the low rates of oocyte maturation and high-quality embryos. The mRNA levels of PTX3 were positively correlated with the oocyte maturation rate (*r* = 0.448, *P* < 0.05, Fig. 2g).

### BMP6 suppresses the expression of PTX3 in KGN and hGL cells

**Fig. 3:**
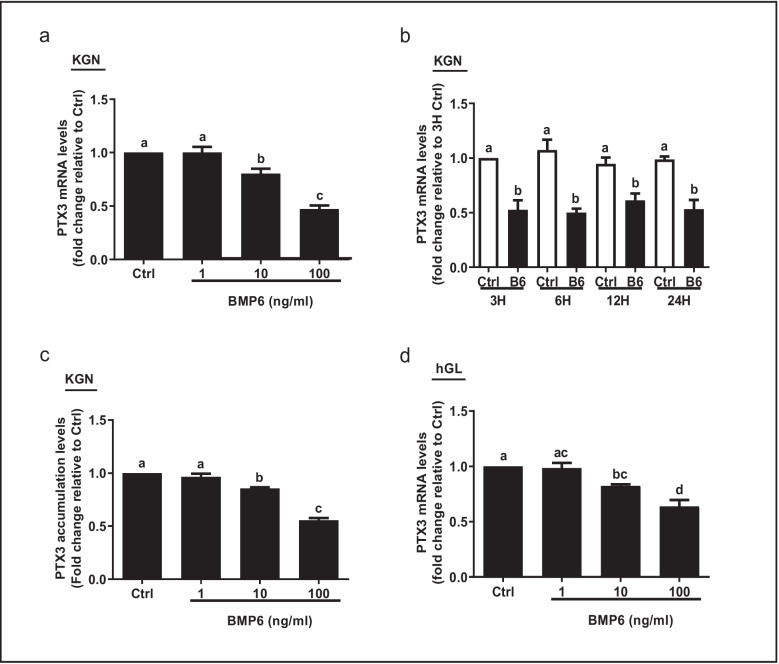
The effects of BMP6 on the expression of PTX3 in KGN and hGL cells. **a** KGN cells were treated with vehicle control or different concentrations (1, 10, or 100 ng/mL) of BMP6 for 12 h, and the mRNA levels of PTX3 were examined using RT-qPCR. **b** KGN cells were treated with 100 ng/mL BMP6 for 3, 6, 12, or 24 h, and the mRNA levels of PTX3 were examined using RT-qPCR. **c** KGN cells were treated for 24 h with vehicle control or different concentrations (1, 10, or 100 ng/mL) of BMP6, and the accumulated protein levels of PTX3 were measured using an enzyme immunoassay. **d** The primary hGL cells were treated with different concentrations (1, 10, or 100 ng/mL) of BMP6 for 6 h, and the mRNA levels of PTX3 were examined using RT-qPCR. The results were expressed as means ± standard errors of at least three independent experiments, with different letters indicating statistically significant differences (*P* < 0.05)

To investigate the effect of BMP6 on the expression of PTX3 in the human ovary, we used recombinant human BMP6 to treat KGN cells. The results showed that treatment with different concentrations (1, 10, or 100 ng/mL) of BMP6 for 12 h significantly decreased the mRNA levels of PTX3 in a concentration-dependent manner using in vitro KGN cell culture (Fig. 3a). We further treated KGN cells with 100 ng/mL BMP6 for different durations (3, 6, 12, or 24 h), and the time course experiments demonstrated that the suppressive effects of BMP6 on the mRNA levels of PTX3 started at 3 h and persisted until 24 h after treatment (Fig. 3b). Similarly, the Western blot analysis results revealed that BMP6 treatment for 24 h decreased the protein levels of PTX3 in a concentration-dependent manner in KGN cells (Fig. 3c). To confirm the physiological relevance of the results obtained from the immortalized cell line, we used primary hGL cells isolated from follicular fluid samples obtained from patients undergoing IVF. Similar to the results obtained from KGN cells, the level of PTX3 mRNA was significantly decreased in primary hGL cells after treatment with different concentrations of BMP6 (1, 10, or 100 ng/mL) for 6 h (Fig. 3d).

### The BMP type I receptors ALK2/ALK3, but not ALK6, mediate the BMP6-induced downregulation of PTX3 in KGN cells

**Fig. 4:**
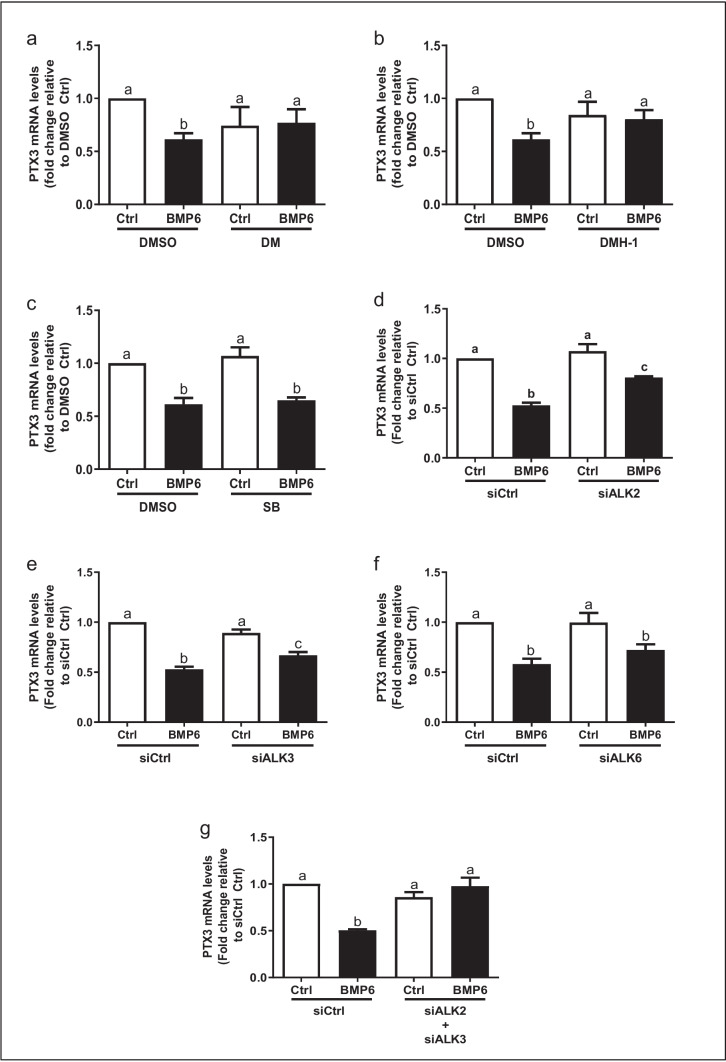
ALK2 and ALK3 type I receptors are required to mediate BMP6-induced downregulation of PTX3 expression in KGN cells. **a–c** KGN cells were pretreated with dimethyl sulfoxide (DMSO), 10 μM DM (**a**), 0.25 μM DMH-1 (**b**), or 10 μM SB431542 for 1 h, and cells were then treated with 100 ng/mL BMP6 for another 6 h. The mRNA levels of PTX3 were examined using RT-qPCR. **d–g** KGN cells were transfected with siCtrl, siALK2 (**d**), siALK3 (**e**), siALK6 (**f**), or combined siALK2 and siALK3 (**g**) for 24 h, and cells were then treated with 100 ng/mL BMP6 for another 6 h. The mRNA levels of PTX3 were examined using RT-qPCR. The results are expressed as means ± standard errors of at least three independent experiments. Different letters indicate statistically significant differences (*P* < 0.05)

Several BMP type I receptors (activin receptor-like kinases or ALKs) have been shown to transduce BMP signaling [30, 31], whereas several kinase inhibitors have been shown to inhibit the activity of ALKs. For instance, DMH-1 selectively inhibits ALK2/3, dorsomorphine (DM) inhibits ALK2/3/6, and SB431542 inhibits ALK4/5/7 [32, 33]. Our results showed that pretreatment with DM (10 μM) or DMH-1 (0.25 μM) for 1 h reversed the suppressive effect of BMP6 on PTX3 mRNA levels in KGN cells (Fig. 4a and b). However, pretreatment with 5 μM SB431542 did not have this effect (Fig. 4c). To further determine which ALKs were involved in this process, a siRNA-based inhibition approach was conducted to knock down the type I receptors (ALK2, ALK3, or ALK6). Previous studies have verified that transfection of cells with siRNA targeting ALK2, ALK3, or ALK6 for 48 h significantly reduced the mRNA levels of specific ALKs [19]. Specifically, the results showed that knockdown of ALK2 or ALK3 partially reversed the suppressive effect of BMP6 on the mRNA levels of PTX3 (Fig. 4d and e), whereas knockdown of ALK6 had no such effect (Fig. 4f). Notably, the combined knockdown of ALK2 and ALK3 completely reversed the suppressive effect of BMP6 on the expression of PTX3 (Fig. 4g). These results indicate that the type I receptor ALK2 or ALK3 is required to mediate the BMP6-induced downregulation of PTX3 expression in KGN cells.

### BMPR2 and ACVR2A are required to mediate the suppressive effect of BMP6 on the expression of PTX3 in KGN cells

**Fig. 5:**
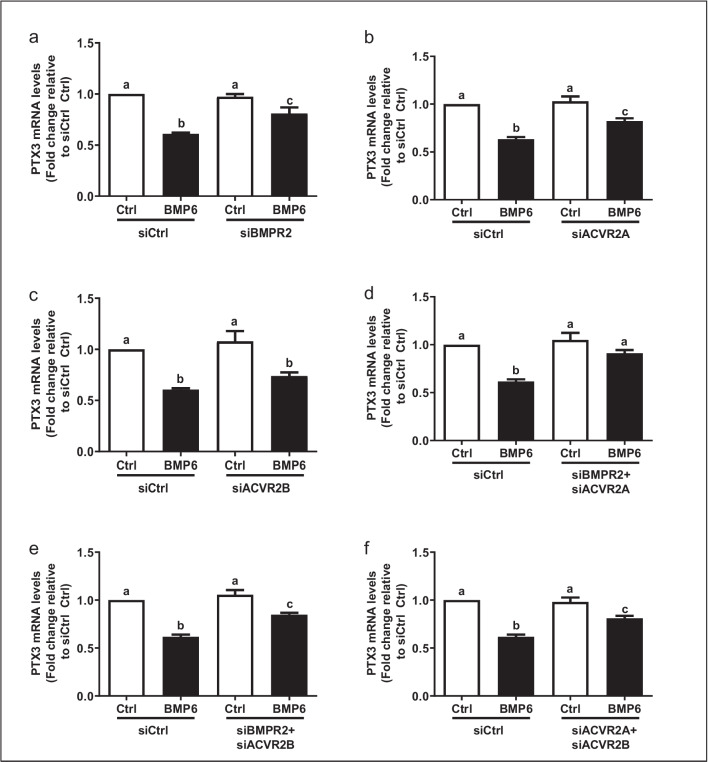
BMPR2 and ACVR2A type II receptors are required to mediate the BMP6-induced downregulation of PTX3 expression in KGN cells. **a–c** KGN cells were transfected with siCtrl, siBMPR2 (**a**), siACVR2A (**b**), or siACVR2B (**c**) for 48 h, and cells were then treated with 100 ng/mL BMP6 for another 6 h. The mRNA levels of PTX3 were examined using RT-qPCR. **d–f** KGN cells were transfected with siCtrl, combined siBMPR2 and siACVR2A (**d**), combined siBMPR2 and siACVR2B (**e**), or combined siACVR2A and siACVR2B (**f**) for 48 h. Cells were then treated with 100 ng/mL BMP6 for another 6 h, and the mRNA levels of PTX3 were examined using RT-qPCR. The results are expressed as means ± standard errors of at least three independent experiments. Different letters indicate statistically significant differences (*P* < 0.05)

Bone morphogenetic protein receptor type II (BMPR2), activin receptor type IIA (ACVR2A), and activin receptor type IIB (ACVR2B) are the functional type II receptors implicated in BMP-induced cellular signaling [30]. A similar siRNA approach was used to determine which type II receptor (BMPR2, ACVR2A, or ACVR2B) mediates the BMP6-induced signaling in KGN cells [33]. The results showed that knockdown of BMPR2 and ACVR2A partially reversed the BMP6-induced downregulation of PTX3 mRNA (Fig. 5a and b). However, knockdown of ACVR2B did not have this effect (Fig. 5c). In addition, we found that the simultaneous knockdown of BMPR2 and ACVR2A completely reversed the BMP6-induced downregulation of PTX3 mRNA (Fig. 5d). However, either concomitant knockdown of BMPR2 and ACVR2B (Fig. 5e) or concomitant knockdown of ACVR2A and ACVR2B (Fig. 5f) did not have this effect. These results indicate that the type II receptors BMPR2 and ACVR2A, but not ACVR2B, are required to mediate the suppressive effect of BMP6 on the expression of PTX3 in KGN cells.

### ALK2/ALK3 type I receptors and BMPR2/ACVR2A type II receptors mediate BMP6-induced phosphorylation of SMAD1/5/8 in KGN cells

**Fig. 6:**
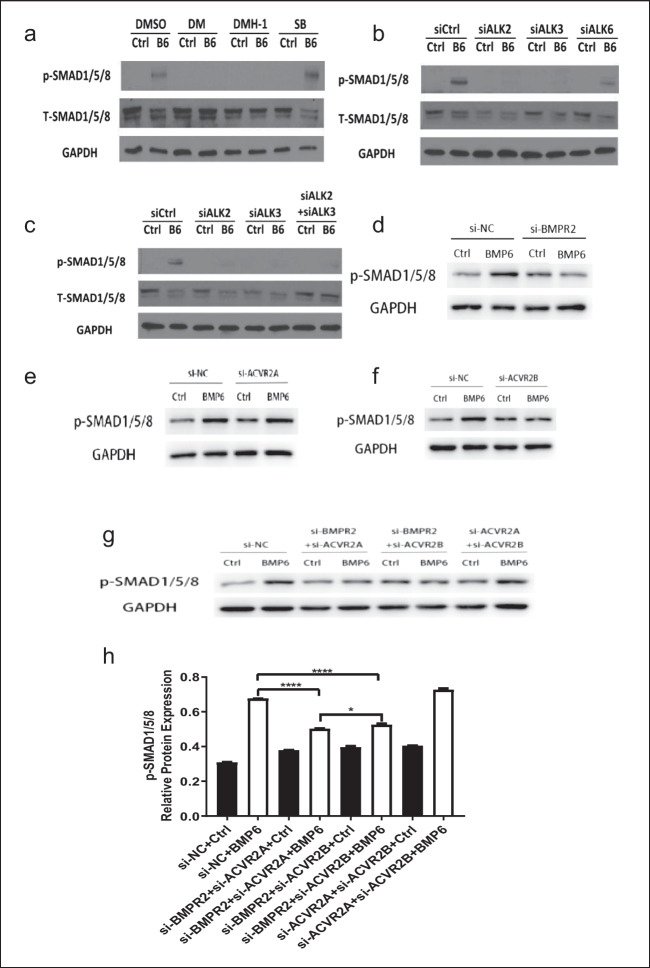
ALK2/ALK3 type I receptors and BMPR2 type II receptor mediate BMP6-induced phosphorylation of SMAD1/5/8 in KGN cells. **a** KGN cells were pretreated for 1 h with DMSO, DM (10 µM), DMH-1 (0.25 µM), or SB431542 (10 µM), and cells were then treated with 100 ng/mL of BMP6 for another 6 h. The levels of phosphorylated SMAD1/5/8 protein levels were examined using Western blot analysis. **b** KGN cells were transfected with siCtrl, siALK2, siALK3, or siALK6 for 24 h, and cells were then treated with 100 ng/mL of BMP6 for another 1 h. The phosphorylated SMAD1/5/8 protein levels were examined using Western blot analysis. **c** KGN cells were transfected with siCtrl, siALK2, siALK3 or combined siALK2 and siALK3 for 24 h, and cells were then treated with 100 ng/mL of BMP6 for another 1 h. The phosphorylated SMAD1/5/8 protein levels were examined using Western blot analysis. **d–f** KGN cells were transfected with siCtrl, siBMPR2 (**d**), siACVR2A (**e**), or siACVR2B (**f**) for 48 h, and cells were then treated with 100 ng/mL of BMP6 for another 6 h. The phosphorylated SMAD1/5/8 protein levels were examined using Western blot analysis. **g–h** KGN cells were transfected with siCtrl, combined siBMPR2 and siACVR2A, combined siBMPR2 and siACVR2B, or combined siACVR2A and siACVR2B for 48 h, and cells were then treated with 100 ng/mL BMP6 for another 6 h. The phosphorylated SMAD1/5/8 protein levels were examined using Western blot analysis

Previous studies have suggested that BMP6 may induce the activation of downstream SMAD1/5/8 proteins [34]. To further determine which receptors were involved in BMP6-induced SMAD1/5/8 phosphorylation, we treated KGN cells with type I receptor inhibitors or knocked down the aforementioned type I and type II receptors using siRNA to analyze phosphorylated SMAD1/5/8 protein levels using Western blot analysis. Our results showed that pretreatment with DM or DMH-1 significantly lessened BMP6-induced increases in phosphorylated SMAD1/5/8 protein levels in KGN cells. However, pretreatment with SB431542 did not have this effect (Fig. 6a). In addition, knockdown of ALK2, ALK3, or combined knockdown of ALK2 and ALK3 significantly reversed the BMP6-induced increase in phosphorylated SMAD1/5/8 protein levels, whereas knockdown of ALK6 did not have this effect (Fig. 6b and c). Interestingly, knockdown of BMPR2, but not ACVR2A or ACVR2B, reversed the BMP6-induced increase in phosphorylated SMAD1/5/8 protein levels (Fig. 6d to f). Our results further showed that the combined knockdown of BMPR2 and ACVR2A or combined knockdown of BMPR2 and ACVR2B reversed the BMP6-induced increase in phosphorylated SMAD1/5/8 protein levels, while the combined knockdown of BMPR2 and ACVR2A reversed the increase in phosphorylated SMAD1/5/8 protein levels to a greater extent, indicating the important role of BMPR2 and ACVR2A in regulating SMAD1/5/8 activity (Fig. 6g and h).

### SMAD1 and SMAD5 mediate the suppressive effect of BMP6 on PTX3 expression in KGN cells

**Fig. 7:**
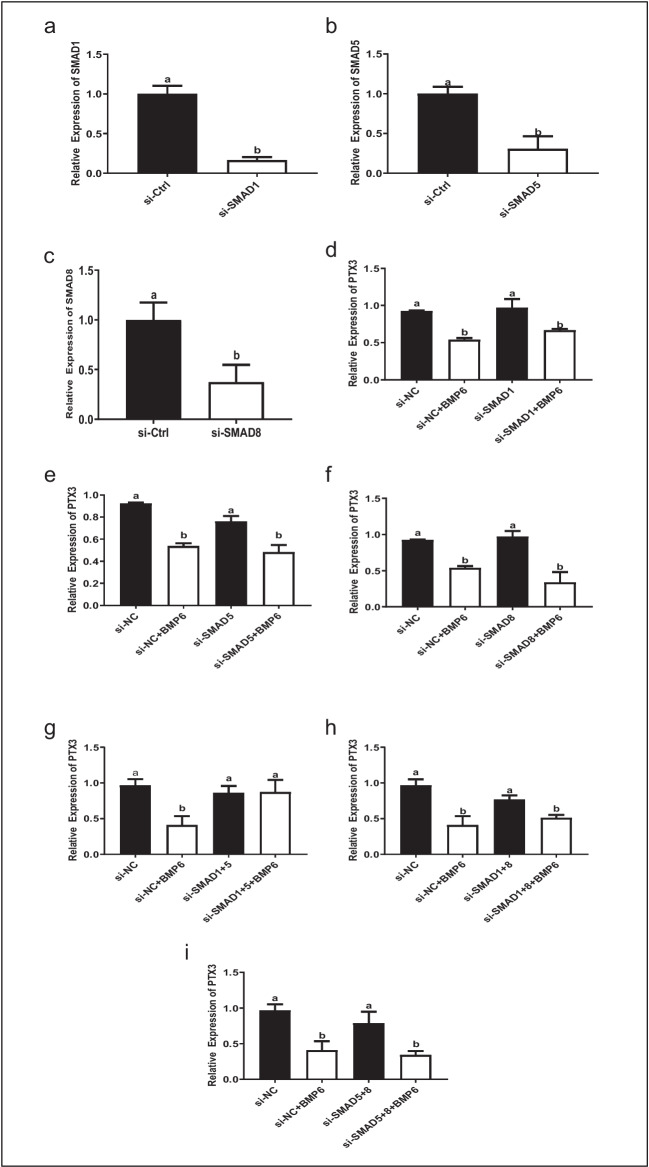
SMAD1 and SMAD5 mediate the suppressive effect of BMP6 on PTX3 expression in KGN cells. **a–c** KGN cells were transfected with 25 nM siCtrl, 25 nM SMAD1 siRNA (siSMAD1) (**a**), 25 nM SMAD5 siRNA (siSMAD5) (**b**), or 25 nM SMAD8 siRNA (siSMAD8) (**c**) for 24 h. The mRNA levels of SMAD1, SMAD5, and SMAD8 were examined using RT-qPCR. **d–f** KGN cells were transfected with 25 nM siCtrl, 25 nM siSMAD1 (**d**), 25 nM siSMAD5 (**e**), or 25 nM siSMAD8 (**f**) for 24 h, and cells were then treated with 100 ng/mL BMP6 for another 6 h. The mRNA levels of PTX3 were examined using RT-qPCR. **g–i** KGN cells were transfected with 25 nM siCtrl, concomitant 25 nM siSMAD1 and siSMAD5 (**g**), concomitant 25 nM siSMAD1 and siSMAD8 (h), or concomitant 25 nM siSMAD5 and siSMAD8 (**i**) for 24 h and then treated with 100 ng/mL BMP6 for 6 h. The mRNA levels of PTX3 were examined using RT-qPCR. The results are expressed as means ± SEMs of at least three independent experiments. Different letters indicate significant differences (*P* < 0.05)

To further determine which SMAD mediates the suppressive effect of BMP6 on the expression of PTX3, specific siRNAs were used to knock down SMAD1, SMAD5, and SMAD8, respectively. The knockdown efficiency of each SMAD was verified using RT-qPCR (Fig. 7a, b, and c). In particular, single knockdown of SMAD1, SMAD5, or SMAD8 did not impact the suppressive effect of BMP6 on the expression of PTX3 in KGN cells (Fig. 7d, e, and f). Interestingly, combined knockdown of SMAD1 and SMAD5 reversed the suppressive effect of BMP6 on the mRNA levels of PTX3 (Fig. 7g). However, combined knockdown of SMAD1 and SMAD8 or SMAD5 and SMAD8 did not have this effect (Fig. 7h and i). These results showed that SMAD1 and SMAD5 were key molecules to mediate the suppressive effect of BMP6 on the expression of PTX3 in KGN cells.

### SMAD4 mediates the BMP6-induced suppressive effect on PTX3 expression in KGN cells

**Fig. 8:**
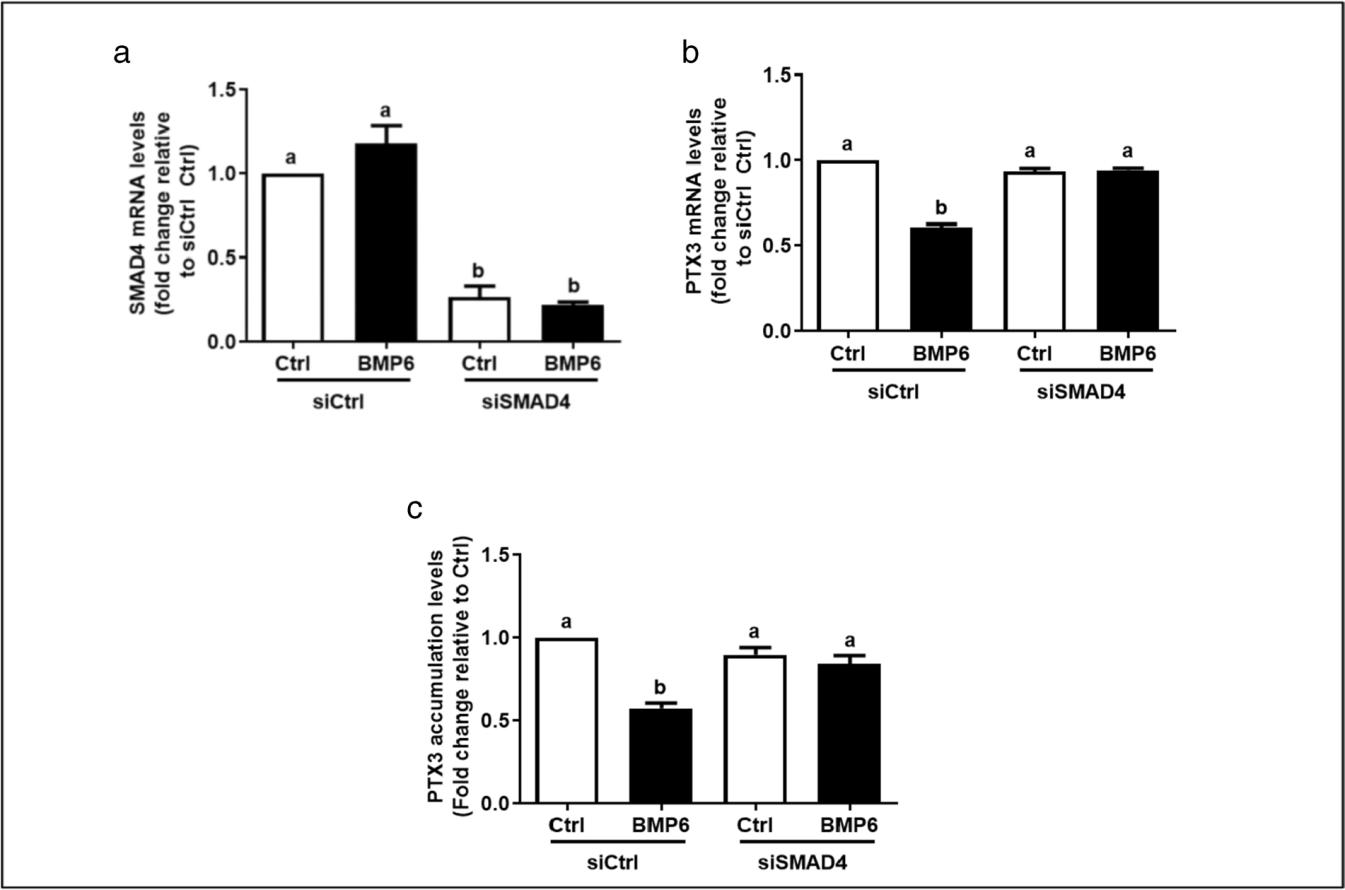
SMAD4 mediates the suppressive effect of BMP6 on PTX3 expression in KGN cells. **a** KGN cells were transfected with 25 nM siCtrl or 25 nM SMAD4 siRNA (siSMAD4) for 24 h, and the mRNA levels of SMAD4 were examined using RT-qPCR. **b** KGN cells were transfected with 25 nM siCtrl or 25 nM siSMAD4 for 24 h, and cells were then treated with 100 ng/mL of BMP6 for another 6 h. The mRNA levels of PTX3 were examined using RT-qPCR. **c** KGN cells were transfected with 25 nM siCtrl or 25 nM siSMAD4 for 24 h, and cells were then treated with 100 ng/mL of BMP6 for another 6 h. The accumulative protein levels of PTX3 were examined using ELISA. The results are expressed as means ± SEMs of at least three independent experiments. Different letters indicate significant differences (*P* < 0.05)

In most tissues, phosphorylated SMAD1/5/8 proteins bind to SMAD4 and translocate to the nucleus to regulate the expression of the related target genes [35]. To further investigate the role of SMAD4 in the BMP6-induced downregulation of PTX3 expression, we knocked down SMAD4 using a specific siRNA. The knockdown efficiency and specificity of siSMAD4 were examined using RT-qPCR (Fig. 8a). Notably, knockdown of SMAD4 completely reversed the suppressive effect of BMP6 on PTX3 mRNA levels in KGN cells (Fig. 8b). Similarly, knockdown of SMAD4 completely reversed the suppressive effect of BMP6 on accumulative PTX3 protein levels in KGN cells (Fig. 8c).

## Discussion

PCOS is one of the main causes of ovulatory dysfunction infertility. The development of assisted reproductive technology has solved the reproductive problems of patients with PCOS to some extent. However, patients with PCOS still face challenges. Although patients with normal ovarian reserves can obtain more follicles, fewer oocytes have good capacities. Of the two groups of patients included in this study, patients with PCOS showed relatively poor follicular maturation ability. Because the oocytes of patients with PCOS did not fully acquire the ability to mature, they were more likely to lead to follicular development stagnation and ovulation disorders [36]. High quality oocytes often have the ability to undergo meiosis, further maturation, and form embryos that can be used for transplantation; they may even form high-quality embryos after fertilization, resulting in a higher likelihood of pregnancy [37]. The relevant results were confirmed in the clinical outcomes of the two groups of patients included in this study.

In addition, in the late stage of follicular development, mural granulosa cells, cumulus granulosa cells, and oocytes work together to induce cumulus expansion, thereby forming mature and capable oocytes. In the ovary, PTX3 is involved in the process of cumulus expansion and is considered to be a marker gene for the developmental potential of oocytes [38]. BMP6 is also considered to be involved in the formation of hyaluronic acid, which is an important substance in the process of cumulus expansion. Considering the pathological state of follicular development disorders in patients with PCOS, we believed that PTX3 and BMP6 may play an important role in the occurrence and development of PCOS. Through PCR experiments on follicular fluid and cumulus granulosa cells in patients with PCOS, we found high levels of BMP6 expression and low levels of PTX3. Interestingly, cell experiments on hGL cells and KGN cells showed that BMP6 had an inhibitory effect on PTX3 expression. To our knowledge, this study is the first to explore the levels of BMP6 and PTX3 in the ovaries of women with PCOS. A possible inhibitory relationship between BMP6 and PTX3 was discovered. Pan et al. found that non-obese women with PCOS had higher levels of PTX3. They believed that PTX3 levels were positively correlated with low-grade inflammatory status in patients with PCOS [39], which differed from the results obtained in our study. Pan et al. suggested that the time interval between hCG injection and oocyte retrieval, as well as BMI, may be important factors affecting the expression levels of PTX3 in the follicular fluid of patients with PCOS. In our study, no difference was identified in BMIs between the PCOS group and the control group. The difference in BMIs between the two groups of patients included in the study may be an important factor affecting the expression of PTX3. In addition, based on the detection of PTX3 levels in follicular fluid obtained during ovarian stimulation therapy, we also tested the levels of PTX3 and BMP6 in primary hGL cells, and the results were consistent with the expression trend in follicular fluid. However, future large-scale, multicenter clinical studies are still needed to deeply explore the relationship between PTX3, BMP6, and clinical pathological features of PCOS.

In this study, we further demonstrated through Spearman correlation analysis that BMP6 was negatively correlated with oocyte maturity and high-quality embryo formation, while PTX3 was positively correlated with oocyte maturity. The relatively high expression of BMP6 and the low expression of PTX3 in patients with PCOS may have a common or subtle interaction, leading to a decrease in oocyte quality and embryonic development potential. In vitro experimental results also demonstrated that BMP6 downregulated the expression of PTX3 in hGL cells and KGN cells. Previous studies suggested that gene mutations in BMP and its receptors, or interruptions in BMP-mediated signaling, may lead to reproductive disorders [40]. Therefore, we can speculate that increased BMP6 activity in the ovaries of patients with PCOS might inhibit the expression of PTX3 through specific pathways, leading to oocyte development arrest and ovulation disorders.

Given the potential role of BMP6 in the pathogenesis of ovulation dysfunction, a comprehensive understanding of the molecular mechanisms underlying the response of PTX3 to BMP6 in cells is crucial for developing treatment strategies for patients with PCOS. Similar to the TGF-β family, BMP induces cellular response signal transduction by interacting with serine/threonine kinase receptors bound to type I and type II membranes [41, 42]. Although BMP6 plays a crucial role in human fertility, the cellular receptors that mediate BMP6 biological activity are largely unknown. Previous studies have confirmed that ALK type I receptors and BMPR2, ACVR2A, or ACVR2B type II receptors may mediate BMP-induced phosphorylation of SMAD1/5/8. After phosphorylation and activation, SMADs bind to SMAD4 and translocate to the nucleus to regulate target gene expression [33, 43]. To understand the role of these two receptor types in the molecular regulatory mechanism of BMP6 on PTX3 expression, we conducted a series of experiments. Our results indicate that the addition of DM (ALK2/ALK3/ALK6 inhibitor) or DMH-1 (ALK2/ALK3 inhibitor) completely reversed the increase in SMAD1/5/8 phosphorylation induced by BMP6 and the inhibition of PTX3 expression, indicating that ALK2 or ALK3 may be involved in BMP6-mediated cell activity. Using siRNA based inhibition methods, we further confirmed that ALK2 and ALK3 are functional type I receptors that mediate BMP6 induced cell activity in human granulosa cells. Using the same method, our results suggest that BMPR2 or ACVR2A may be involved in BMP6-induced cellular effects, indicating that BMPR2 and ACVR2A are functional type II receptors in the molecular regulatory mechanism of BMP6 on PTX3 expression. However, BMPR2 may be the main type II receptor for BMP6-induced cellular activity.

Receptor activated SMADs or receptor regulated SMADs (R-SMADs), including SMAD 1, SMAD 5, and SMAD 8, are essential for BMP-mediated cellular activity [44]. Using similar inhibition methods, we demonstrated that SMAD1 and SMAD5 were functional signaling molecules that mediated BMP6 to inhibit PTX3 expression in KGN cells. Our results also indicated that SMAD4 was an indispensable mediator in the downstream signaling pathway induced by BMP6, which led to inhibition of PTX3 expression. However, this study is only a preliminary result obtained from in vitro cell experiments and needs to be validated through clinical trials. The study of key receptors in the BMP6 regulatory pathway of PTX3 may contribute to the development of a new approach for treating PCOS.

In summary, our research findings reveal the physiological role and potential molecular mechanisms of BMP6 and PTX3 in regulating ovarian function and ovulation, which may be related to the pathogenesis of PCOS. However, shortcomings and limitations remain in this study. For example, our study included a small number of patients and detected the expression levels of related factors in cumulus granulosa cells and follicular fluid after drug-induced ovarian stimulation, which may affect the correlation between BMP6, PTX3, and PCOS. In addition, our study did not consider the subgroups of patients based on BMI and cannot rule out the impact of BMI on the experimental results. Future in-depth research is necessary to better understand the expressions of PTX3 and BMP6 genes at different stages of follicular development in the ovaries of patients with PCOS.

## Data Availability

All data generated or analyzed during this study are included in this published article.
